# Paediatric Residents and Fellows Ethics (PERFEct) survey: perceptions of European trainees regarding ethical dilemmas

**DOI:** 10.1007/s00431-021-04231-8

**Published:** 2021-08-24

**Authors:** M. C. den Boer, A. Zanin, J. M. Latour, J. Brierley

**Affiliations:** 1 Division of Neonatology, Leiden University Medical Center, Albinusdreef 2, Leiden, The Netherlands; 2 Department of Medical Ethics and Health Law, Leiden University Medical Center, Albinusdreef 2, Leiden, The Netherlands; 3 Department of Women’s and Children’s Health, University of Padua, Via Giustiniani, 3-35128 Padua, Italy; 4 School of Nursing and Midwifery, Faculty of Health, University of Plymouth, Plymouth, PL4 8AA UK; 5 Nursing Department, Hunan Children’s Hospital, Changsha, China; 6 Paediatric Bioethics Centre, University College London, NIHR Great Ormond Street Hospital Biomedical Research Centre, London, UK

**Keywords:** Ethics, Training, Paediatrics, Medical education, Perceptions, Physicians

## Abstract

With an increasingly complex healthcare environment, ethics is becoming a more critical part of medical education. We aimed to explore European paediatric trainees’ experiences of facing ethical dilemmas and their medical ethics education whilst assessing their perceptions of ethical dilemmas in current and future practice. The Young Sections of the European Academy of Paediatrics and European Society of Paediatric and Neonatal Intensive Care developed an explorative online survey covering demographics, ethical dilemmas faced and ethics training. The survey was made available in nine languages from November 2019 to January 2020 via newsletters and social media. Participants (*n* = 253) from 22 countries, predominantly female (82%) and residents (70%), with a median age of 29-years, completed the survey. The majority (58%) faced ethical dilemmas monthly or more frequently. Most ethics training was received by ethics lectures in medical school (81%) and on the job (60%). A disagreement between the healthcare team and patient/family was the most frequently faced moral dilemma (45%); the second was withholding/withdrawing life-prolonging measures (33%). The latter was considered the most challenging dilemma to resolve (50%). Respondents reported that ethical issues are not sufficiently addressed during their training and wished for more case-based teaching. Many have been personally affected by moral dilemmas, especially regarding withholding/withdrawing life-prolonging measures, and often felt inadequately supported.

*Conclusion*: Paediatric trainees face many moral issues in daily practice and consider that training about managing current and future ethical dilemmas should be improved, such as by the provision of a core European paediatric ethics curriculum.

**Table:** 

**What is Known:** *• Paediatric services are becoming more complex with an increase in ethical dilemmas asking for rigorous training in ethics.* *• Ethics training is often lacking or covered poorly in both pre- and postgraduate medical education curricula.* *• Existing ethics training for European paediatric trainees is haphazard and lacks standardisation.*	
**What is New:** *• The PaEdiatric Residents and Fellows Ethics (PERFEct) survey provides insight into the European paediatric trainees’ views regarding ethical dilemmas in their current and future practice.* *• European paediatric trainees report a lack of ethics training during paediatric residency and fellowship.* *• This study provides content suggestions for standardised medical ethics training for paediatric trainees in Europe.*

**Supplementary information:**

The online version contains supplementary material available at 10.1007/s00431-021-04231-8.

## Introduction

Paediatricians face many ethically challenging situations. Some are unique to paediatrics, as they involve specific aspects such as child growth and development or parent and child decision-making [1]. Decisions of limiting life-prolonging treatment, therapeutic disagreements with families and end-of-life (EOL) discussions are some of the most prevalent ethical dilemmas (ED) that paediatricians face [2]. In such situations, paediatric residents and fellows are often the frontline providers caring for children and their families, but little is known about their experience or training.

Although ethics training is widely included in undergraduate medical programs [3], it is often absent in resident curricula [4], including for paediatrics. Reports from the US identified a number of issues with ethics training including a lack of ethics training for paediatric residents [5] and programmes not meeting the actual ethical challenges of paediatric residents [6]. Moreover, paediatricians exposed to formal ethics training during paediatric residency or fellowship reported a lack of ethics knowledge, supporting the need for more targeted educational interventions [7]. A literature review, including 96 studies, confirmed several barriers to ethics training among paediatric residents and recommended regular case-based ethics training session [3].

Some European paediatric residency programmes include ethics training as part of their core curriculum [8–11]. However, the impact of undergraduate and postgraduate ethics education on both trainee’s knowledge, especially regarding the ethical challenges they face, and clinical practice is unknown. Furthermore, ethics curricula that exist do seem inconsistent across European countries. A needs assessment and content formulation for paediatric ethics training across Europe would be helpful. With this exploratory study, we therefore aimed to assess (1) the ethics training experience of European paediatric trainees, (2) their perceptions of ethical dilemmas (EDs) in their current and future practice, and (3) their educational needs.

## Methods

The European Academy of Paediatrics (EAP) and the European Society of Paediatric and Neonatal Intensive Care (ESPNIC) are actively involved in developing European educational programs for paediatricians. Their Young sections collaborated in this study to identify paediatric trainees’ views, needs and current training experience regarding ethical dilemmas (EDs).

### Design

For our survey, we used a pre-existing exploratory survey produced by the Ethics Strategic Advisory Group of EAP. This survey was developed by experts in the field of paediatric ethics. We adapted this survey in order to make it more applicable to paediatric trainees and piloted with seven international paediatric trainees, reflecting differing target populations and nationalities with a shared ethical milieu specific to paediatric trainees. The survey was translated from English into eight other languages: Dutch, French, German, Italian, Latvian, Portuguese, Russian and Spanish.

The Checklist for Reporting Results of Internet E-Surveys (CHERRIES) [12] has been used to report the study. The study was conducted according to the principles of the Declaration of Helsinki (Brazil 2013) and the General Data Protection Regulation (E.U. 2016/679), approved by the scientific committees of EAP and ESPNIC and reviewed by the Ethics Review Committee of the Leiden University Medical Center (Reference nr. C19.058).

### Participants and recruitment

The target population was European paediatric trainees, defined as paediatric residents and fellows. A convenience sampling strategy was used, with participants recruited through young ESPNIC and young EAP via the society member newsletters and social media channels. The survey was available on the online platform SurveyMonkey from November 2019 to January 2020. Participants were told that completion of the survey demonstrated consent to participate, have responses analysed and for publication of pooled anonymous responses. There was no compensation or reimbursement.

### Survey

The survey’s scope was related to EDs in current and future paediatric practice; general ethics training, specifically paediatric ethics training content, and confidence in knowledge about ethics. Furthermore, respondents were questioned about the received support when facing EDs. The survey was sub-divided into five sections: (1) demographics (9 items), (2) education (3 items), (3) EDs (12 items), (4) most recent ethical challenge (7 items), and (5) training and education (10 items). Throughout the survey, EDs were listed as follows: withholding/withdrawing life-prolonging measures, disagreement amongst the healthcare team, disagreement between the healthcare team and the patient/family, the refusal of treatment by the patient/family, patient autonomy, legal issues, who has parental responsibility, religious or cultural issues, experimental treatment, truth-telling, professional conduct and other. In various sections, perceptions of trainees were queried on scales. At the end of the survey, three open-ended questions were included to provide suggestions for improving ethics training during paediatric traineeship. The complete survey is available at Electronic Supplement 1.

### Analysis

Data were obtained and recorded without identifiers, protected by Secure Sockets Layer (SSL) encryption, and analysed in aggregate form. Responses from paediatric consultants, trainees rounding outside of Europe and participants that did not complete the first three sections of the survey were excluded from the analysis. Quantitative data were analysed using IBM-SPSS Statistics 25 for Windows. To determine percentages, we classified responses that were left blank as missing. Categorical data are presented as *n* (%). Perceptions of trainees were queried on a scale from 0 to 10 (strongly disagree to strongly agree) and presented as median (IQR). The Independent *t* test and *χ*^2^ test set at *p* < 0.05 were used to compare residents and fellows and compare gender. The *χ*^2^ test and one-way ANOVA test set at *p* < 0.05 were used to compare trainees rounding at various departments (general paediatrics, Paediatric Intensive Care Unit (PICU), Neonatal Intensive Care Unit (NICU), others and by region. For comparison by region, countries were classified into three regions (northern Europe, central Europa and southern Europe), in concordance with the ETHICUS study’s division [13]. We performed Content Analysis [14, 15] of open-ended questions using the qualitative software programme Atlas.ti (v.8.4). Data were first reviewed by open coding; subsequently, two researchers analysed data in categories (MCdB, AZ). During consensus meetings with three researchers (MCdB, AZ, JML), the categories were classified into two themes: (1) training forms and (2) training topics.

## Results

In total, 327 surveys were returned, of which 18 were excluded because respondents were consultants (*n* = 15), outside Europe (*n* = 1), or data about the country was missing (*n* = 2). Data of 56 respondents were excluded from analysis because Sects. 1–3 were not fully completed. Consequently, data of 253 respondents were included in the analysis of trainees’ perceptions regarding EDs in current practice. Subsequently, data from another 36 participants were excluded as they did not complete Sect. 5 (EDs in paediatric training). As a result, data of 217 respondents were included in the analysis of EDs in paediatric training.

### Demographics

Participant characteristics are presented in Table 1. Most respondents were female (82%) and residents (70%), with a median age of 29 years. Respondents were rounding in 22 European countries. Most respondents were rounding at the NICU (20%), general paediatrics (19%) or the PICU (14%).

**Table 1:** Demographics(*n* = 253)

Female *n (%)*	208 (82)
Age *median (IQR)*	29 (27–32)
Resident *n (%)*	179 (70)
Country *n* (%)	
Austria	12 (5)
Switzerland	21 (8)
Spain	22 (9)
France	29 (12)
Italy	24 (9)
Latvia	19 (8)
Netherlands	14 (5)
Portugal	43 (17)
Slovenia	15 (6)
Ukraine	25 (10)
UK	13 (5)
Other	16 (6)
Department *n* (%)	
Anaesthesia	10 (3)
Cardiology	6 (3)
E.R	8 (3)
General paediatrics	49 (19)
Infectious diseases	5 (3)
NICU	52 (20)
PICU	36 (14)
Primary care	9 (4)
Other	29 (12)
Unknown	49 (19)
Religion *n* (%)	
Catholic	92 (37)
Agnostic/Atheist	105 (42)
Christian Orthodox	27(10)
Islamic	2 (1)
Jewish	2 (1)
Other	25 (10)

### EDs in current practice

More than half of the respondents (58%) reported facing EDs monthly or more frequently. The most encountered EDs were disagreement between healthcare professionals (HCPs) and patient/family (45%), withholding/withdrawing life-prolonging measures (33%) and disagreement amongst the healthcare team (31%) (Table 2). EDs concerning withholding/withdrawing life-prolonging measures were statistically less faced by trainees working in general paediatrics (*p* < 0.001), by residents (*p* = 0.001) and by trainees from southern European countries (*p* = 0.026) (Electronic Supplement 2). EDs that trainees considered most challenging to resolve were withholding/withdrawing life-prolonging measures (50%), refusal of treatment by the patient/family (42%) and disagreement between the healthcare team and patient/family (41%) (Table 2). Fellows reported significantly more difficulties with solving EDs concerning disagreement amongst the healthcare team (*p* = 0.021), female trainees with solving EDs concerning truth-telling (*p* = 0.021) and trainees rounding in southern European countries with solving EDs concerning withholding/withdrawing life-prolonging measures (*p* = 0.020).

**Table 2:** Ethical dilemmas (%)

	Most frequently faced ethical dilemmas (*n* = 253)	Most difficult ethical dilemma to resolve (*n* = 253)	Most occurring ethical dilemma in 20 years (*n* = 217)	Mostly addressed topics (*n* = 217)	More training desired (*n* = 217)
Withholding/withdrawing	33	50	63	52	59
Disagreement team	31	25	43	24	53
Disagreement patient/family	45	41	63	38	63
Refusal treatment	18	42	51	36	56
Patient autonomy	9	12	40	24	41
Legal issues	7	17	48	35	54
Parental responsibility	8	8	36	17	29
Religious/cultural	15	26	44	26	43
Experimental treatment	4	8	38	13	34
Truth-telling	8	27	31	19	48
Professional conduct	6	6	24	21	41
None	0	0	0	3	0

**Fig. 1:**
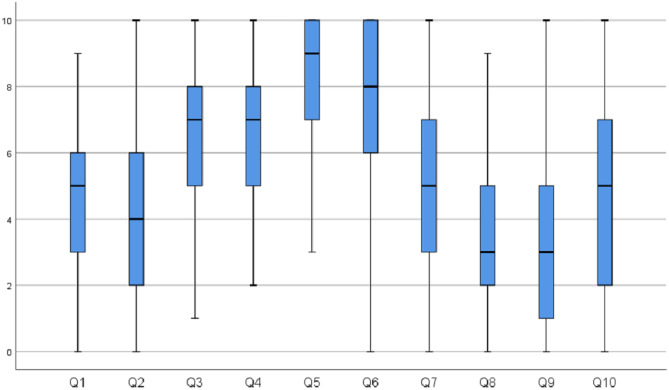
Perceptions of trainees

Although EDs occurred frequently, they were rarely discussed with an ethical committee (Table 3). Trainees reported the need to involve parents and children in the decision-making regarding EDs. Furthermore, trainees said that they did not feel actively involved in the decision-making themselves (Fig. 1).

**Table 3:** Background training (*n* = 217)

Ethics education *n (%)*	
Ethics lectures in med school	175 (81)
Required reading	33 (15)
Required courses in residency	40 (18)
Required courses in fellowship	7 (3)
Experience on the job	130 (60)
Intensive course	9 (4)
Certificate program	2 (1)
Mentoring	8 (4)
Other	17 (8)
None	16 (7)
Ethical dilemma presented to ethical committee *n (%)*	
Yes	65 (30)
No	138 (64)
My hospital does not have an ethical committee	14 (6)
Audits at department *n (%)*	
Yes	48 (22)
No	105 (48)
Not sure	64 (29)
Participated in simulation *n (%)*	
Yes	68 (31)
No	149 (69)

### EDs in paediatric training

The most frequent ethics training reported was ethics lectures in medical school (81%) and experience on the job (60%) (Table 3). Fellows significantly reported more on the job experience compared to residents (*p* = 0.012). Only 18% of respondents reported participation in required courses in residency, and 11% of fellows reported participation in required courses during their fellowship. Thirty one per cent of respondents participated in ethics simulations, with significantly more fellows reporting participation in simulation (*p* = 0.044). Paediatric trainees reported intermediate confidence in ethics. Furthermore, respondents reported that current and future EDs are not adequately addressed during their training (Fig. 1).

Most reported EDs addressed during paediatric training were withholding/withdrawing of life-prolonging measures (52%), disagreement between the healthcare team and the patient/family (38%), refusal of treatment by patient/family (36%) and legal issues (35%) (Table 2). However, trainees reported that existing ethics training did not meet their educational needs (Fig. 1). Fellows significantly desire more training than residents in parental responsibility (*p* = 0.027) and professional conduct (*p* = 0.049). Female trainees wanted considerably more training in professional conduct (*p* = 0.002).

Many trainees (78%) reported being affected personally by EDs faced during training and sadly highlighted a lack of support at that time, especially from their trainer (56%) (Table 4). Fellows said they had been personally affected by EDs more often than residents (*p* = 0.013), especially in situations of withholding/withdrawing life-prolonging measures (*p* = 0.045).

**Table 4:** Personal affection and support (*n* = 217)

Ethical dilemmas that personally affected me *n (%)*	
Withholding/withdrawing	81 (30)
Disagreement team	63 (23)
Disagreement family	61 (23)
Refusal treatment	47 (17)
Patient autonomy	14 (5)
Legal issues	17 (6)
Parental responsibility	16 (6)
Religious/cultural	23 (8)
Experimental treatment	4 (1)
Truth-telling	27 (10)
Professional conduct	19 (7)
Other	1 (0)
None	60 (22)
Missing	1 (0)
Most support received from *n (%)*	
Colleague Trainer Peer Partner Counsellor No one Other Missing	67 (31) 39 (18) 41 (19) 37 (17) 1 (0) 18 (8) 7 (3) 7 (3)
Support desired from *n (%)*	
Colleague Trainer Peer Partner Counsellor No one Other Missing	64 (29) 120 (56) 38 (18) 17 (8) 38 (18) 21 (10) 6 (3) 11 (5)
Dedicated reference person for ethically challenging situations *n (%)*	
Yes No Not sure Missing	75 (34) 85 (39) 56 (26) 1 (0)

### Qualitative data

One hundred sixty respondents answered the three open-ended questions. The 22 categories were synthesised to the themes “training forms” and “training topics” (Table 5). Many respondents reported that they would like any/more mandatory ethics training (*n* = 61). The most suggested training activities were case-based training (*n* = 56) and simulation (*n* = 45). Trainees identified 32 topics for additional training, with legal issues (*n* = 23), the interaction between parents and the healthcare team (*n* = 13), ethical theory (*n* = 8) and cultural/religious aspects (*n* = 6) the most frequent.

**Table 5:** Qualitative data (*n *= 160)

Training forms	
Audits/debrief	5
Case-based	56
Conversation training	11
Discussions	27
Involvement	17
Lectures	22
Mentoring	8
Seminars	27
Simulation	45
Training (general)	61
Other	16
Training topics	
Autonomy	3
Cultural/religious	6
End-of-life	4
Interaction parents/patient	13
Interaction team	3
Legal aspects	23
Palliative care	3
Patient rights	4
Theory	8
Truth-telling	3
Other	24

## Discussion

This study assessed the perceptions of paediatric trainees on EDs in their current and future practice. More than half reported frequent ethical challenges, such as disagreement between healthcare teams and patient/family, withholding/withdrawing life-prolonging measures and disagreement among healthcare team members. Many did not feel actively involved in ethical decision-making processes. Respondents said that current and future EDs are not adequately addressed during their training and wished for more case-based training and simulation. Worryingly, many respondents did not feel adequately supported when they were involved in decisions about withholding/withdrawing treatments in children, identifying the need for better support from their supervisors.

Attention to education in ethics and professionalism among medical students and postgraduate doctors is increasing, and consequently, some improvement in ethics training in the postgraduate training is in progress. However, in our study, only 18% of paediatric residents and 11% of paediatric fellows reported participation in any mandatory ethics courses required by university curricula during their training programme. Furthermore, trainees reported only intermediate confidence in ethics knowledge. Our study thus shows that there is still an area for improvement in ethics education for paediatric trainees.

The results of our study provide new information and perspectives on ethics training in paediatrics. For example, respondents reported considerable effort is concentrated on delivering education and discussions about withholding/withdrawing life-prolonging measures. This focus seems appropriate given trainees said that EDs related to this area caused them the most concern, and ethics training is essential for the most frequent and concerning topics encountered [3]. However, other EDs were also often experienced but rarely covered in training, including refusal of treatment, truth-telling and legal issues. Efforts should undoubtedly be made to address these topics in ethics training.

Using EDs that are being encountered in daily practice is a sensible starting point to determine relevant ethics training priorities. Using in-depth interviews, Rosenbaum et al. [16] described five categories of EDs for residents in internal medicine, including truth-telling, respecting patients’ wishes, preventing harm, managing the limit of one’s competence and dealing with disagreement within the team and the perception of an inappropriate performance of others. In our study, these topics were also recognised as fundamental in ethics training, but the discussion about withholding/withdrawing life-prolonging measures seemed the most relevant. This may be explained by the broader availability of life-sustaining therapies and the fact that different cultures and countries deal differently with this [9]. Another major issue raised by study respondents was disagreement with families and refusal of treatments. Effective communication with patients and families is the foundation of the therapeutic relationship [17]. It is a crucial component in daily paediatrics but especially vital when decisions about the use of life-sustaining treatment are needed. Effective communication can be taught in various ways, for instance, by using simulation [18–21], which our respondents also explicitly suggested.

Both in paediatric and neonatal intensive care, professionals are commonly challenged by questions about their practice and the EDs they face. These can lead professionals to experience moral distress [22–25]. In the daily practice of paediatric and neonatal intensive care, moral distress is frequent and relates to several difficult situations dealing with the patients’ outcome and management but also with difficulties in communication among team members. Various strategies can help professionals to cope with moral distress, including organizational, personal and administrative actions [26]. These strategies may lead to a redistribution of workload, mutual support among professionals and the development of techniques to cultivate open communication and questioning within the multidisciplinary team. The aim of an adequate ethics training program should also be to teach, discuss and promote coping strategies for moral distress, meanwhile maintaining the focus on the patient and acting with moral courage and good communication in an environment of mutual respect [26]. In our survey, many trainees reported a lack of support from their supervisors when facing ethical challenges. This may also be a possible resource of coping for trainees. As such, supervisors and senior colleagues may possibly be empowered in their personal ethical education path, introducing also programs to learn to recognize, prevent and mitigate moral distress among residents [23], in order to have both proactive and reactive strategies and to offer a possible roadmap for attending physicians to help their residents navigate moral distress.

The disconnection between existing ethics education and what paediatric trainees consider they need offers educational institutions and medical societies an opportunity to plan, improve and deliver ethics education. Trainees’ recommendations seem an ideal starting point, and so more case-based training, direct workplace supervision, focussed teaching by consultants and the use of simulation scenarios are required. Interestingly, ethics simulation is reported in nursing practice, but no reports are available in medical residents training to our knowledge. Norrena [27] and Wilt [28] reported some experiences of high-fidelity ethics simulation scenarios developed and implemented in the paediatrics course of nursing programs. Simulation scenarios may be used to analyse very challenging points of discussion, such as disagreements with families expressing vaccine hesitancy, empowering clinicians and improving awareness of their own biases toward vaccine-refusing families, allowing them to become acquainted with their potential role in enforcing good practices [29]. Simulation scenarios may furthermore contribute to developing coping and communication skills [30–32]. Moreover, simulation contributes to the development of junior doctors’ autonomy, empowering them in their role as decision-makers.

As both physicians and patients can freely move within Europe, CESMA — the European body tasked with monitoring training assessment in Europe — has been involved in the development of a board certification exam for all European residents in Pediatrics [33]. Similarly, we suggest that an agreed European paediatric core ethics curriculum would be beneficial, allowing shared understanding, approved core knowledge and training development across centres. However, doing so can be challenging, as European countries have different stances on ethical issues. Therefore, a European core program should not impose a common specific position on sensitive ethical issues but should be aimed at supporting physicians that face ethical dilemmas to reflect on these dilemmas, both within the context of their own cultural and moral background, as well as within the context of the patients they treat [34]. Furthermore, we argue that a European core program should also include training in legal issues. Although many child healthcare law topics will be nation-specific, generic training in the principles of consent and confidentially, sources of supra-national law and the law surrounding the most challenging EDs encountered will benefit paediatric trainees.

It was concerning that many trainees reported a lack of support from their supervisors when they faced ethical challenges. However, it seems reasonable to speculate that this might be due to senior paediatricians having low confidence, familiarity and even core knowledge in this area, given they have probably received little core training in ethics. Most supervisors will have less ethics education than current trainees report receiving, given that there are limited postgraduate education opportunities in this area. Therefore, to accompany any development of European paediatric trainee ethics curricula, senior physician programs should also be considered. We recommend that the European training boards and scientific societies should meet this demand for shared Ethics Educational Programs across European countries by developing a shared Paediatric ethics curriculum and, taking advantage of the increasing opportunities related to remote learning, provide and promote international meetings, webinars, case discussions and round table debates as a complementary resource that can be integrated to the local modules. Furthermore, further research is required to develop a European paediatric core ethics curriculum. 

### Limitations

Our survey study has several limitations, including the small number of participants compared to the total number of European paediatric trainees. Furthermore, the findings may not represent all European countries, as the numbers of respondents from several countries are relatively low (e.g. only 2 Germans). Moreover, missing data and questions left unanswered due to our survey’s lengthiness may bias findings if those with missing data are systematically different, or inefficient statistical estimates occur due to the loss of information. Finally, like all survey studies, the PERFEct survey had standardised questions; due to the heterogeneity of European Paediatric trainees, it is difficult to ask anything other than very general questions in a questionnaire that a broad range of people will understand. In this sense, a survey may be less valid than different national data collection methods, but this method allowed us to examine the topic across countries comprehensively.

## Conclusion

Paediatric trainees frequently experience EDs in their practice but report both a lack of ethics training and involvement in the management of EDs. Ethics training should be included in all European paediatric curricula, whether general or specialist, to improve future paediatric consultants’ core knowledge and skills in dealing with complex ethical situations and their ability to deliver training in this nuanced area.

Rather than individual specialities developing this in silos, this could be readily achieved by creating a specific shared European paediatric ethics curriculum, standardised European case-based (simulation) training and associated proactive live training and support for trainees by paediatric consultants during the management of ethical challenges. Further research is needed to understand the gaps in ethics knowledge that may help educators responsible for postgraduate medical education review or revise the ethics curriculum accordingly.

## Supplementary information

Below is the link to the electronic supplementary material.

Supplementary file1 (DOCX 18 KB)

## Data Availability

Unidentified data are available upon reasonable request.

## Abbreviations

CHERRIES: Checklist for Reporting Results of Internet E-Surveys
EoL: End-of-life
ED: Ethical dilemmas
EAP: European Academy of Paediatrics
ESPNIC: European Society of Paediatric and Neonatal Intensive Care
HCPs: Healthcare providers
NICU: Neonatal Intensive Care Unit
PERFEct: PaEdiatric Residents and Fellows Ethics
PICU: Paediatric Intensive Care Unit
SSL: Secure Sockets Layer
