# Supplementary material for: Paediatric Residents and Fellows Ethics (PERFEct) survey: perceptions of European trainees regarding ethical dilemmas
Source: Eur J Pediatr. 2021 Aug 24;181(2):561–70. doi: 10.1007/s00431-021-04231-8 (PMC8821074; doi:10.1007/s00431-021-04231-8)
Supplement: Supplementary file 1 — Supplementary file1 (DOCX 18 KB) [file 431_2021_4231_MOESM1_ESM.docx]

| **Checklist for Reporting Results of Internet E-Surveys (CHERRIES)** | |  |
| --- | --- | --- |
| ***Item Category*** | ***Checklist Item*** | ***Explanation*** |
| **Design** |  |  |
|  | Describe survey design | An explorative online survey, aimed at European Paediatric Residents and Fellows, convenience sample |
| **IRB (Institutional Review Board) approval and informed consent process** |  |  |
|  | IRB approval | Approved by IRB, LUMC (C19.058) |
|  | Informed consent | Participants were informed that participants consented to participate in this study by completing the survey.  Participants were informed that by completing the survey, they consented to the analysis and publication of pooled unidentified responses. |
|  | Data protection | Data were obtained and recorded without identifiers, protected by Secure Sockets Lay Secure Sockets Layer (SSL) encryption, and analysed in aggregate form. |
| **Development and pre-testing** |  |  |
|  | Development and testing | We developed an exploratory survey and piloted it with seven international paediatric trainees, reflecting differing target populations and nationalities with a shared ethical milieu specific to paediatric trainees. The survey was translated from English into eight other languages: Dutch, French, German, Italian, Latvian, Portuguese, Russian, and Spanish. |
| **Recruitment process and description of the sample having access to the questionnaire** |  |  |
|  | Open survey versus closed survey | Open survey |
|  | Contact mode | By mail and via social media |
|  | Advertising the survey | By mail and via social media |
| **Survey administration** |  |  |
|  | Web/E-mail | Web survey |
|  | Context | SurveyMonkey |
|  | Mandatory/voluntary | Voluntary |
|  | Incentives | No incentives |
|  | Time/Date | Nov 2019- Jan 2020 |
|  | Randomization of items or questionnaires | Questionnaire |
|  | Adaptive questioning | No adaptive questioning |
|  | Number of Items | Differt |
|  | Number of screens (pages) | 5 |
|  | Completeness check | No completeness check, answers could be left open |
|  | Review step | Back button |
| **Response rates** |  |  |
|  | Unique site visitor | N/A |
|  | View rate (Ratio of unique survey visitors/unique site visitors) | N/A |
|  | Participation rate (Ratio of unique visitors who agreed to participate/unique first survey page visitors) | N/A |
|  | Completion rate (Ratio of users who finished the survey/users who agreed to participate) | 217/327 |
| **Preventing multiple entries from the same individual** |  |  |
|  | Cookies used | N/A |
|  | IP check | N/A |
|  | Log file analysis | N/A |
|  | Registration | N/A |
| **Analysis** |  |  |
|  | Handling of incomplete questionnaires | Sub-analysis for incomplete questionnaires |
|  | Questionnaires submitted with an atypical timestamp | N/A |
|  | Statistical correction | N/A |
